# Medicine in Vienna and Paris

**Published:** 1887-03

**Authors:** W. S. Caldwell

**Affiliations:** Paris


### FOREIGN CORRESPONDENCE.

### MEDICINE IN VIENNA AND PARIS.

*To the Editor of the* JOURNAL AND EXAMINER.

*Dear Sir:*

The man in busy practice with little time to read, if he takes a deep interest in his profession, often feels a desire to break off from the monotonous routine of his labors, and visit the great centers of medical learning, see and hear the men who teach, and make himself acquainted with their manner of treating their patients.

In thirty years of active professional work this is the only desire that I have gratified.

While the few random notes that this letter contains will perhaps be nothing new to the man who stays at home and by diligently reading his text-books keeps himself abreast of the times in his profession, still I believe there is an advantage in seeing men treat their patients as compared with that of reading their writings, not only in the matter of having the subject illustrated by a living patient who is examined before you, and whom you may examine yourself, but I believe further that men really often treat their cases themselves very differently from the manner in which they recommend the same to be done when they write a text-book, or an article for publication in a medical periodical. In what I shall say I shall purposely abstain from reporting or discussing unusual or extraordinary cases, confining myself to those that fall under our care at every turn in actual practice.

Most American physicians whom one meets here are young men just from the colleges, and they are too often only interested in such cases as a man would see only occasionally in a lifetime. They are eager to see Bibroth resect the pylorus for cancer, and are constantly presenting their cards to Pèan to get a chance to see operations, that they never ought to attempt to perform.

But I am even more disgusted with some of the lady physicians who come here to Paris.

They will be pushed and jostled amid the great throng that follow the surgical service of Gillan at Hotel Dieu; sit on a back seat with an opera glass in hand to see Pèan amputate a man's leg at the hip joint. But go to the children's hospital, where Simon holds his clinic, and one finds not a single one of them there.

The manner of teaching medicine in Germany is somewhat different from that practiced in France.

In Vienna, for example, a patient is wheeled into theamphitheatre, a couple of students are called down and requested to examine the case and give a diagnosis.

After they have finished their examinations, the professor reviews the case and corrects any errors that may have been made. He discusses the case fully, you are informed from time to time as to its progress, and when it dies you are expected to attend the autopsy.

You get but little chance to examine the cases at the bedside unless you take one of the private courses, given by one of the assistants.

Here in Paris there are so many hospitals and so many good men who give bedside instruction, that one can follow them and personally examine every case of interest.

While at the bedside the professor tells you which case he proposes to make the subject of his morning lecture; he then, after examining the case in your presence, giving its most salient points, repairs to the lecture-room and discusses the disease more fully.

Looking over a period of twenty years, at which date I made my first visit to the continent, I find many changes in the treatment of diseases, especially those of an acute character.

These changes are of two kinds: first, a tendency to go back to the practice of our fathers in the matter of the administration of mercury, and the more frequent practice of blood-letting; and second, a more complete and absolute expectancy. While the late discoveries in bacteriology have shed new light upon our means of diagnosis, unfortunately it has added but little to our stock of knowledge as far as therapeutics is concerned.

There is another peculiarity about the prescriptions of most continental physicians that is perhaps worthy of note, and that is, that they scarcely ever combine two remedies in the same recipe, or even give the patient two active remedies at the same time.

Arterial sedatives, such as aconite, veratrum viride, and the like, so commonly prescribed with us, I have never heard even mentioned, much less prescribed, during my present stay in Europe.

We smile with pity at the laity who swallow quack nostrums and make millionaires of the Ayers, the Jaynes and the Warners, and yet we physicians of the West, at least, allow a few enterprising drug houses that manufacture beautifully colored tasteless elixirs and pleasant syrups, said to contain the medicinal properties of roots and herbs of whose real action we know little or nothing, and the best that can be said of them is that they are generally inert. I say that we allow ourselves to be humbugged on a scale that is gigantic, and for which there is no apology except that like the non-professional world we are easily gulled, or never stop to seriously investigate the subject. But I had forgotten that I was writing up medical matters in Europe and not in America.

Bamberger, of Vienna, who is one of the most scientific men in Europe, I believe, is a perfect skeptic as to the beneficial effects of drugs in the treatment of acute diseases. Though an old man and rather feeble in health, he never flags in his interest at the bedside, examines his cases with the most pains-taking exactness, and if they die, follows them to the dead-house and views with an anxious eye every cut made by the scalpel that is either to prove or disprove the diagnosis made during the life of the patient.

Ten years ago he was using in typhoid fever the cold baths after the manner and teachings of Liebermeister. Now he uses cold baths much more sparingly, in fact, only when the temperature reaches 40° C. He then uses them every three or four hours until the heat of the body is reduced to 38.5° C. In general, however, he prefers to lessen the bodily heat by repeated cold or tepid sponging.

The only medicine he gave a series of patients I saw him treat was seven grains of quinine at 1 and 5 P.M. The large doses of quinine, recommended by Barthelow and other American authors, are universally condemned here. Though such quantities lessen the temperature, the effect upon the heart's action and nervous system is most disastrous. Nothnagel's treatment of typhoid fever is essentially the same as that adopted by Bamberger, except that he uses the cold baths a little more freely.

In cases where heart-failure becomes a prominent symptom, he gives nitro-glycerin in doses of from .001 to .002 grams three or four times in the twenty-four hours. Great stress is laid on the nourishment of the patient, but only fluids are allowed until the patient is well on the road towards convalescence. Milk and animal broths are exclusively used.

Though a slight albuminuria is nearly a constant symptom of this disease after the 7th to 12th day, an excess of albumen in the urine is a grave symptom.

This should be watched, and when it appears combat it by small doses of digitalis alternated with one-grain doses of calomel.

Here in Paris the treatment of typhoid fever is essentially the same as that in Vienna, except that the cold baths are not generally used.

About 15 grains of quinia is given in the twenty-four hours, divided usually into four doses.

Alcoholic stimulants are used sparingly from the outset, giving the first week about 12 ounces of Bordeau wine, and after that adding a certain amount of brandy. Coffee is given freely for its tonic action upon the heart, and in Paris bread and rice is added to the soup.

In connection with this subject of the treatment of typhoid fever, I have made most thorough inquiry as to the use and their effects of antipyrin and thallin.

Last summer, in an article to the New York *Medical Record*, I gave the opinion of the best men in Germany, as far as I could ascertain, as to the effects of these remedies in this and other febrile diseases. I also gave a review of a very interesting article published in the *Wiener Medizinischer Blätter*, giving an extensive experience with these medicines. Still, I see in nearly every journal I receive from America some one who still treats his cases of typhoid fever with these, in my opinion, most unsafe and dangerous agents.

The writer in the above periodical says that he knows that the remedy has a bad effect in both typhoid fever and pneumonia, and attributes the death of two patients suffering from the latter disease, directly to the administration of antipyrin.

Nothnagel says that while this agent will reduce the temperature of the body to any point desired, it has no effect on the duration of typhoid fever; in fact, its tendency is to prolong the disease—to increase the tendency to hypostatic pneumonia, heart-failure and other untoward symptoms that originate in an enfeebled heart's action, and a general nervous prostration.

Germain Seè, in a late clinical lecture at Hotel Dieu, says:

"Antipyrin in typhoid fever is worse than useless. It never ought to be given.

"In the fever of tuberculosis it will reduce the temperature on the day it is given, but during the apyrexia the patient will sweat more profusely, will feel weaker, and in every way be the worse for the treatment. In this disease attend to the patient's digestion and elimination, and abjure all remedies that produce a profound impression upon the system.

"Never give the remedy for its antipyretic effect, except in a form of fever, the course of which is of short duration and in which there is no ultimate danger from a general anæmia.

"You will find rheumatic patients occasionally who cannot take the salicylate of soda on account of its unpleasant effects upon the stomach or the nervous system.

"If the disease be of an active or sthenic type, you may give the antipyrin with advantage. In certain forms of neuralgia you will find the remedy a good one."

Either at the bedside or in the lecture-room, I have interviewed Jaccoud at Le Pitiê, Professor Peter at the Necker, Professor Potain at Le Charitê, and Professor Dieulafoy at the School of Medicine, and they all tell one story, and that is, give *no antipyrin in typhoid fever.*

If I shall be able in this article to convince the readers of this journal, or even a small part of them, that this remedy is one that never ought to be given in any form of fever that has a natural tendency to run a protracted course, I shall certainly have rendered a most valuable service to their patients.

### BRIGHT'S DISEASE.

Professor Dieulafoy, who has lately been appointed to a professorship of practice in the School of Medicine, is a young man, and one of the finest teachers at the bedside and in the lecture-room that I have ever heard. He opened his course after the holidays by a series of lectures on diseases of the kidneys.

He says in relation to Bright's disease that the nice points that authors try to make as to the particular form of lesion that exists in any given case will all fail you generally at the bedside. That, in fact, all the different lesions are often found in the same kidney, and as to which one predominates is a point that can only be determined with certainty by a post mortem examination. His treatment all resolved itself into a milk diet and blood-letting.

He insists on blood-letting whenever the patient presents any symptoms that portend uræmic convulsions.

Professor Jaccoud's treatment is the same, in addition to which he is now using the inhalation of oxygen gas.

He says that the number of casts or the amount of albumen in a given case he considers of less importance in a prognostic point of view than the specific gravity of the urine. You may think the patient better because there is less albumen in the urine, but unless one finds an improvement in the weight of the urine, he will be disappointed. He insists on blood-letting, and that early if the case be a grave one.

Professor Peter relies mostly on a milk diet in his treatment, but gives small doses of digitalis on alternate days.

He says that the tincture or fluid extract of digitalis is never reliable.

He advises an infusion or a decoction, but he prefers the former. In Vienna they also use the remedy only prepared in this way. In the latter city they usually give one-grain doses of calomel on alternate days, using the digitalis on the day when the calomel is not given.

In Professor Simon's wards in the children's hospital there are always a large number of cases of Bright's disease.

Professor Simon says that there are two diseases that children tolerate much better than adults, which fact should always be taken into account when formulating a prognosis; and these are cardiac and renal affections.

The patients in this hospital range in age from 3 to 14 years. He begins the treatment with digitalis, using an infusion. He continues this only three or four days. He watches the pulse, and if it begins to grow more frequent, he suspends the remedy for a few days and then begins again. He keeps the bowels open with small doses of calomel. He washes the surface twice a day with hot alcohol, and if the patient cannot tolerate the digitalis by the stomach, he adds that to the alcohol used for the bath. He uses a hot bath twice a day, and envelops the body in carded wool for two hours after each bath. In case of convulsions he uses an injection of hydrated chloral. He applies cups to the spine, and draws blood in proportion of the age and strength of the patient.

PLEURITIC EFFUSION.

Among the working-classes here, who are constantly exposed to the vicissitudes of a damp winter climate, with their houses generally insufficiently heated, they have in the hospitals here a large number of cases of serous effusion into the pleuritic cavity. In the service of Professors Jaccoud and Peters I have witnessed the management of quite a number of cases of this kind. The former is a firm believer in the efficacy of early tapping.

As soon as the effusion reaches an amount to exceed 32 ounces, he uses the aspirator and draws off the fluid. On the other hand, Professor Peter only taps the patient when the fluid fills the pleural cavity to such an extent as to interfere with respiration, or deranges the circulation by the displacement of the heart. He relies mainly in his treatment on blisters to the chest, using them of large size and changing from one locality to another on the chest walls.

He sometimes gives the iodide of potassium in five-grain doses three times a day; but blisters, combined with a generous diet and plenty of wine, are his main reliance.

Professor Jaccoud, to a few of his patients, gives quinine in three-grain doses three times a day. But his great remedy in these cases is *milk*. He now has a patient in the wards to whom he gives one gallon of milk every twenty-four hours, and to whom he allows no other diet.

He claims that this manner of treatment is far better than giving medicines, such as the iodide of potassium and the like; that the milk has a double action: that is, to nourish the patient, and to act powerfully upon the kidneys, causing absorption of the effused fluid.

Like women in the matter of bonnets, we doctors follow fashions in rushing after new ideas in the treatment of diseases, and when we take a start in a certain direction are all too much inclined to run in the same rut.

The mania now in Paris is the milk-treatment. Though, like most other hobbies, it is probably often ridden without sense or judgment, it has one redeeming feature in its favor, which cannot always be said of medicines proper, and that is, that it is a harmless kind of craze.

Professor Dieulafoy, in his opening lecture at the School of Medicine, advocated in most eloquent terms the practice of early puncture in cases of pleuritic effusion. He says that whenever the amount of the fluid in the chest is as much as thirty-two ounces, it is one's duty to warn the patient and the family that the case may terminate fatally at any moment, and cites a number of examples in his practice to prove the truth of this assertion.

PNEUMONIA.

In the broncho-pneumonia of children the treatment here is ipecacuanha to the extent of vomiting the patient occasionally, the use of the bromide of potassium to quiet the cough, and the free use of alcohol. No opium is given. Mild forms of counter-irritation are applied to the chest.

In croupous-pneumonia the treatment is expectant and alcohol is used, though Professor Jaccoud gives tartarized antimony in the early stages when the patient is robust.

INTESTINAL OBSTRUCTION.

A man aged 37 years was admitted into Hotel Dieu, in the service of Professor Gillaux, having all the symptoms of intestinal obstruction, which had existed for eighteen hours. Gillaux said that he was unable to determine the exact nature of this obstruction, and, as the case was not yet sufficiently grave to warrant an abdominal section, he would put the man to bed, give a large hypodermic of morphine, and apply a mild faradic current to the abdomen for twenty-four hours, unless the patient's symptoms grew worse. He accordingly applied a large wet sponge to the abdomen, and on top of this he applied one electrode, and the other opposite over the spinal cord. This was kept up for twenty-four hours, at the end of which time the bad symptoms had all disappeared, and the man made a good recovery. Gillaux said that this is his usual treatment of such cases, and he had often been rewarded with as equally brilliant results as in the present case.

### M. PASTEUR.

During my stay in Paris I have visited at intervals M. Pasteur's institute, and although he is out of health himself and is spending the winter in the south of France, I have witnessed a large number of patients treated by his method of anti-rabic inoculation.

I have also watched with great interest the medical journals here, and listened with attention to the men who lecture at the School of Medicine, in order to form an idea of the sentiment of the profession here in France as to the merits of this, the greatest discovery or the most plausible humbug of this 19th century.

In the medical press the subject is but little discussed, and in speaking of M. Pasteur the faculty often refer to his great researches in bacteriology, but say little or nothing, pro or con, as to the merits of his claimed anti-hydrophobic discoveries. Still I am inclined to believe that the majority of the profession here are believers in Pasteurism, and all of them have perfect faith in his personal honesty and integrity.

Early in January, Dr. Vulpian made a report before the Academy of Medicine, giving the statistics of M. Pasteur's institute up to December 31st, 1886.

Up to that time there had been treated there 2,682 cases. Of these there were 518 who had been bitten by animals only supposed to be mad. The balance, that is 2,164 cases were bitten by animals known to be affected by hydrophobia.

Of these 29 had died up to that date, or 1.34 per centum. Previous to M. Pasteur's treatment statistics show that 16 persons out of every 100 bitten by rabid animals had died of rabies.

This result, of course, according to Dr. Vulpian and M. Pasteur's admirers in general, stamps him as one of the greatest benefactors of his race.

During the latter part of the summer and early autumn of 1886, a larger number of cases treated at the institute had died than during any corresponding period of time before. This decided M. Pasteur to use a stronger virus for his injections, and he accordingly instituted *la Mèthode intensive.*

Up to this time things went on quietly, and M. Pasteur treated, from month to month, a larger number of cases, and collected from all quarters money with which to found a great institute, to more thoroughly carry out his work. If there was much opposition to his method, it did not show itself upon the surface.

Early in January last, Professor Peter, who is one of the first physicians in Paris, read before the Academy of Medicine the history of a case that he had lately treated in connection with a fellow practitioner, and which case had been treated at the Pasteur Institute. This patient died fourteen days after the last treatment of a form of rabies called the paralytic form of the disease, a mode of death which uniformly follows when animals are inoculated with the virus of hydrophobia. Professor Peter described in detail the patient's symptoms, and avowed openly that in his opinion the patient died of the treatment given, and not from the bite of the supposed rabid animal.

This report produced a most profound sensation, not only in professional ranks, but the secular press, taking sides either for or against M. Pasteur, discussed the subject at great length. Professor Peter, though attacked most violently by the friends of M. Pasteur, is a brave little fellow, and defends his position with a tenacity and vigor that are terrible. Two weeks after this, in a clinical lecture before us at the Necker, he spoke for two hours, in substance as follows:

"On December 31 we had admitted into our service here a man 30 years of age with high fever, severe headache and pains in his loins. No diagnosis was made out at first. Four days later the patient broke out with the small-pox. We have had five cases of small-pox follow this contamination in the wards. As soon as it was known that the first patient had small-pox all the other inmates were vaccinated, that is, four days after they were infected. One of these patients died of small-pox, and all the others who had the disease had, at the same time, the vaccine or cow-pox, each disease running its regular typical course in each patient, but being modified by the circumstances that the two forms of disease existed at the same time.

These cases illustrated a law long since recognized; that is, for preventive inoculation to be of any account it must be practiced before the patient is exposed to the disease for which the preventive vaccination is practiced. Therefore, for the inoculation of M. Pasteur to be of any account, according to all the laws of analogy in such cases, it ought to be practiced on the patients before they are exposed to the hydrophobic poison.

Within the last few days I have treated a patient aged 14 years that had been bitten on the finger by a cat. The animal was not suspected at the time to have had hydrophobia, but as two horses belonging to the child's parents six days later died of this disease, it was suspected that the cat was mad.

At this date, that is six days after the boy was bitten, he was taken to M. Pasteur and was treated by *la Mode intensive* for twelve days.

Six days after the last treatment, the patient was taken, early in the morning, with a pain in his back and a general malaise. A physician was called who told the parents that the boy had simply a lumbago and would soon be all right. Two hours later the physician was called back to see his patient, whom he found suffering from complete paraplegia. Professor Peter saw the patient at this time, who grew gradually worse, the paralytic symptoms extending rapidly upward and producing death at the end of twenty-six hours.

This patient had no convulsions, had no difficulty in swallowing water, and, in fact, had no true hydrophobic symptoms.

Professor Peter claimed most strenuously that this patient, as well as the one he had reported at the Academy, had died of the treatment he had received by M. Pasteur. He says that the friends of inoculation claim that the paralytic form of the disease sometimes occurs where no treatment has been used. I say that there have been recorded nine cases of paralytic hydrophobia in 200 years; while M. Pasteur's treatment has given us seven out of eight of the last deaths that have occurred after having been inoculated by him."

Professor Peter not only attacked M. Pasteur's whole plan of treatment but his statistics as well, claiming that the figures given by him did not include all France. That taking France altogether for thirty years prior to Pasteurism the mortality was 30 per annum; that if the same territory be included in the statistics it would give a mortality for 1886 of 42. Since this date Professor Peter says that a report of two other cases have been sent to him, where death occurred in the same manner.

W. S. CALDWELL.

Paris, February, 1887.

